# The Diseases of Females; Including Those of Pregnancy and Childbed

**Published:** 1850-05

**Authors:** 


*The Diseases of Females; including those of Pregnancy and Child-bed.* By Fleetwood Churchill, M. D., Author of the Theory and Practice of Midwifery and Diseases of Infants. *A new American edition revised by the Author.* With the notes of R. M. Huston, M. D., Professor of Materia Medica and General Therapeutics in Jefferson Medical College, &c. Philadelphia: Lea & Blanchard, 1850.

The popularity and acceptableness of Dr. Churchill's works among the profession in this country is now an established fact, of which the rapid sale of his productions is convincing proof. The volume before us appears as the fifth American edition, revised by the author to meet the demand for a new issue; its appearance, therefore, will be hailed by all as an old and valued friend. In casting our eye over its pages, (a criticism upon which has already been presented to our readers on previous editions,) various modifications, alterations, and additions have presented themselves to us, which, we are sure, cannot fail to add to the interest of the volume. In the chapter on Displacements, the subject of retroflexion is introduced for the first time, and a brief notice given of its causes, symptoms, treatment, &c. In the chapter on Encysted Dropsy, also, the history of the operation of ovariotomy is detailed somewhat at length, and the arguments for and against its performance presented, with all the attendant difficulties. The author's conclusions are worthy of attention; he says: "But bearing in mind these difficulties, and making allowances for these drawbacks, I think we may conclude that there are cases in which the operation would be justifiable, and on these grounds: we find the general opinion is against the curability of the disease by medical means; that after a time the patient will die from local disease, or accident, or constitutional disturbance, and that, meantime, she suffers more or less inconvenience; that tapping in almost all cases affords but temporary relief; and that, as far as the limited statistics we possess, it is attended with great danger."

Upon the subject of tapping in encysted dropsies, Dr. W. L. Atlee, of this city, a reliable authority upon this subject, holds the following emphatic language: "Since the year 1823, both in my own and my brother's practice, private and hospital, numerous instances of this operation occurred. I have yet to witness the first case of death in consequence of it, and I have never yet seen serious symptoms arise either from first or other tappings. I have made thein quiry of several experienced surgeons in reference to its dangers, and I have uniformly received a negative reply. I must confess that I was startled by the assertion that 'of first tappings, one-half have been speedily followed by the death of the subject;' a mortality which exceeds that of any of the capital operations, and which, certainly, does not stain the escutcheon of American surgery."* To our minds, the knowledge of this experience conveys the conviction that many cases have been sacrificed to the fear of this operation, that might have been saved by it, had it been timely applied, either as a substitute for the more hazardous operation of gastrotomy, or as a preliminary to it. The same writer, Dr. Atlee, states, that "within the last four years I have treated two cases with success in this way." Both were tapped and treated by medicine, and both are now in the enjoyment of perfect health. The paper which we have thus briefly alluded to, (and which we regret that our space will not allow us to notice more at length,) concludes with a comparison of the statistics of the mortality of the operation for ovariotomy, with those of the mortality following other capital operations, in which it is shown that the chances of life are in favor of gastrotomy. The rate of mortality in operations upon the larger arteries as published by Dr. G. W. Norris, is one in 2.95-96, or 33.45 deaths in 100 cases. While in all the cases of ovariotomy reported, it is 1 in 3 .2-59, or 32.96 cases in 100. Or if we take the results of 78 cases pub-

* Amer. Jour. Med. Sci., new series, April, 1850.

lished since 1845, the proportion of deaths is 26.92 in 100; making the rate of mortality greater in operations upon the large arteries by 25 per cent. than in gastrotomy.

In the diseases of pregnancy, the chapter on Nausea and Vomiting has been re-written, and the question as to the propriety of the induction of premature labor ably discussed. We commend this chapter to the careful attention of our readers.

The work under notice is already too well established in the favor of the profession to need any recommendation of its merits from us. We therefore commit it to their care, with the full assurance that it will meet the same ready welcome so warmly bestowed upon its predecessors.

